# Cognitive behavioural therapy for irritable bowel syndrome: 24-month follow-up of participants in the ACTIB randomised trial

**DOI:** 10.1016/S2468-1253(19)30243-2

**Published:** 2019-09-03

**Authors:** Hazel A Everitt, Sabine Landau, Gilly O'Reilly, Alice Sibelli, Stephanie Hughes, Sula Windgassen, Rachel Holland, Paul Little, Paul McCrone, Felicity L Bishop, Kim Goldsmith, Nicholas Coleman, Robert Logan, Trudie Chalder, Rona Moss-Morris

**Affiliations:** a School of Primary Care Population Sciences and Medical Education, University of Southampton, Southampton, UK; b Centre for Applications of Health Psychology, University of Southampton, Southampton, UK; c Department of Biostatistics and Health Informatics, Institute of Psychiatry, Psychology and Neuroscience, King's College London, London, UK; d Health Psychology Section, Institute of Psychiatry, Psychology and Neuroscience, King's College London, London, UK; e King's Health Economics, Institute of Psychiatry, Psychology and Neuroscience, King's College London, London, UK; f Academic Department of Psychological Medicine, Institute of Psychiatry, Psychology and Neuroscience, King's College London, London, UK; g King's College Hospital, London, UK; h Department of Gastroenterology, Southampton University Hospital, Southampton, UK

## Abstract

**Background:**

Irritable bowel syndrome (IBS) is common, affecting 10–20% of the adult population worldwide, with many people reporting ongoing symptoms despite first-line therapies. Cognitive behavioural therapy (CBT) is recommended in guidelines for refractory IBS but there is insufficient access to CBT for IBS and uncertainty about whether benefits last in the longer term. Assessing Cognitive behavioural Therapy for IBS (ACTIB) was a large, randomised, controlled trial of two forms of CBT for patients with refractory IBS. ACTIB results showed that, at 12 months, both forms of CBT for IBS were significantly more effective than treatment as usual at reducing IBS symptom severity in adults with refractory IBS. This follow-up study aimed to evaluate 24-month clinical outcomes of participants in the ACTIB trial.

**Methods:**

In the ACTIB three-group, randomised, controlled trial, 558 adults with refractory IBS were randomly allocated to receive either therapist-delivered telephone CBT (telephone-CBT group), web-based CBT with minimal therapist support (web-CBT group), or treatment as usual (TAU group) and were followed up for 12 months. Participants were adults with refractory IBS (clinically significant symptoms for ≥12 months despite being offered first-line therapies), recruited by letter and opportunistically from 74 general practices and three gastroenterology centres in London and the south of England (UK) between May 1, 2014, and March 31, 2016. Primary outcome measures were IBS Symptom Severity Score (IBS-SSS) and Work and Social Adjustment Scale (WSAS), assessed in the intention-to-treat (ITT) population with multiple imputation. This study was a non-prespecified naturalistic follow-up and analysis of the participants of the ACTIB trial at 24 months assessing the same outcomes as the original trial. Outcome measures were completed online by participants or a paper questionnaire was posted, or telephone follow-up undertaken. The ACTIB trial is registered with the International Standard Randomised Controlled Trial Number registry, number ISRCTN44427879.

**Findings:**

24-month follow-up of outcomes was achieved for 323 (58%) of 558 participants: 119 (64%) of 186 in the telephone-CBT group, 99 (54%) of 185 in the web-CBT group, and 105 (56%) of 187 in the TAU group. At 24 months, mean IBS-SSS was 40·5 points (95% CI 15·0 to 66·0; p=0·002) lower in the telephone-CBT group and 12·9 points (−12·9 to 38·8; p=0·33) lower in the web-CBT group than in the TAU group. The mean WSAS score was 3·1 points (1·3 to 4·9; p<0·001) lower in the telephone-CBT group and 1·9 points (0·1 to 3·7; p=0·036) lower in the web-CBT group than in the TAU group. A clinically significant IBS-SSS change (≥50 points) from baseline to 24 months was found in 84 (71%) of 119 participants in the telephone-CBT group, in 62 (63%) of 99 in the web-CBT group, and in 48 (46%) of 105 in the TAU group. In total 41 adverse events were reported between 12 to 24 months: 11 in the telephone-CBT group, 15 in the web-CBT group, and 15 in the TAU group. Of these, eight were reported as gastrointestinal related, five as psychological, and six as musculoskeletal. There were no adverse events related to treatment.

**Interpretation:**

At 24-month follow-up, sustained improvements in IBS were seen in both CBT groups compared with TAU, although some previous gains were reduced compared with the 12-month outcomes. IBS-specific CBT has the potential to provide long-term improvement in IBS, achievable within a usual clinical setting. Increasing access to CBT for IBS could achieve long-term patient benefit.

**Funding:**

UK National Institute for Health Research.

## Introduction

Irritable Bowel Syndrome (IBS) is a common chronic gastrointestinal disorder affecting 10–20% of the adult population worldwide, with many people having ongoing symptoms and incurring substantial health costs.^1^, ^2^ Abdominal pain, bloating, and altered bowel habits affect quality of life, social functioning, and time off work.^3^ Currently, clinicians have few options to offer people with refractory IBS. The National Institute for Health and Care Excellence (NICE) guidance^3^ recommends cognitive behavioural therapy (CBT) for patients with refractory IBS symptoms (ie, ongoing symptoms after 12 months despite being offered appropriate medications and lifestyle advice). Published trials of CBT for IBS have reported promising results at 6 months follow-up after randomisation.^4^, ^5^ However, there is still insufficient access to CBT for IBS on the UK National Health Service (NHS) and worldwide. Additionally, there is little evidence of the longer-term outcomes after CBT for IBS, as highlighted in a Cochrane review.^6^

**Research in context**

**Evidence before this study**

We searched the PubMed, PsycInfo, and Cochrane Library from database inception to Jan 7, 2019, without language restrictions for full papers reporting randomised controlled trials, systematic reviews, and meta-analyses with the search terms “irritable bowel syndrome” and “cognitive behavioural therapy”. We excluded trials of adolescents, educational, and group interventions.

Previous research evidence, including several randomised controlled trials, suggests that cognitive behavioural therapy (CBT) is likely to be helpful for irritable bowel syndrome (IBS) in the short term. However, limitations of previous trials include small size, high dropout rates from therapy, and lack of longer-term follow-up. The UK National Institute for Health and Care Excellence guideline for IBS recommends offering CBT for people with refractory IBS but acknowledges that further research is needed. Currently there is insufficient access to CBT for IBS and many patients have no access to psychological therapies for IBS.

The ACTIB trial was, we believe, the largest study of CBT for IBS worldwide to date, recruiting 558 participants with 12-month follow-up. At 12 months, important clinically and statistically significant benefits in IBS symptoms and impact on life were found for both telephone-delivered and web-based CBT interventions compared with treatment as usual.

**Added value of this study**

This follow-up study provides a 24-month, naturalistic follow-up of the ACTIB participants. Participants received no further therapist input but both CBT intervention groups had sustained improvement in the primary and secondary outcomes at 24 months. This improvement was achieved in a clinical setting with relatively modest amounts of therapist contact time.

**Implications of all the available evidence**

This longer follow-up of a large randomised controlled trial adds robust data to the evidence base indicating that IBS-specific CBT has the potential to provide substantial long-term improvement in IBS symptoms, functional impairment, and mood and is achievable within a usual clinical setting. Both the higher-intensity therapist-delivered telephone CBT and the web-based CBT with minimal therapist support can achieve significant clinically important improvements at 24 months. This trial reinforces NICE guidance, which relied on a small evidence base, that CBT for IBS has the potential to benefit patients with refractory IBS and should be made more widely available for this patient group.

Assessing Cognitive behavioural Therapy in IBS (ACTIB) was a three-group, multicentre, randomised, controlled trial (RCT) of two modes of CBT designed specifically for IBS (therapist-delivered, telephone CBT with a patient self-management manual [telephone CBT] and web-based CBT with minimal therapist support [web CBT]) compared with treatment as usual (TAU) alone, in adults with refractory IBS.^7^, ^8^ To our knowledge, it is the largest RCT of CBT for IBS completed thus far and the only one to test the effectiveness of therapist-delivered and web-based CBT in the same trial. The ACTIB trial results showed that telephone CBT and web CBT were significantly more effective than TAU at reducing IBS symptom severity and impact on life at 12 months in adults with refractory IBS.^8^ This result was achieved within the NHS (CBT-trained NHS therapists delivered the interventions).

The aim of the current study was to evaluate longer-term (24 month) clinical outcomes of telephone CBT and web CBT compared with TAU in adults with refractory IBS.

## Methods

### Study design and participants

This study reports a naturalistic 24-month follow-up of participants in the ACTIB trial. The ACTIB trial protocol^7^ and the trial results^8^ at 3 months, 6 months, and 12 months have previously been published.

Participants were people with refractory IBS who were randomly allocated to therapist-delivered telephone CBT (telephone-CBT group), web-based CBT with minimal therapist support (web-CBT group), or TAU (TAU group). ACTIB participants were recruited from 74 primary care general-practice surgeries in the south of England and London (UK), and three secondary-care gastroenterology outpatient clinics in two regions (Southampton University Hospital [Southampton, UK] and Guy's and St Thomas' Hospital Trust, King's College Hospital [London, UK]) between May 1, 2014, and March 31, 2016.

Individuals were eligible if they fulfilled criteria for refractory IBS, defined as fulfilling Rome III criteria for IBS;^9^ reported ongoing clinically significant symptoms according to the IBS Symptom Severity Score (IBS-SSS;^10^ ie, IBS-SSS≥75); had been offered first-line therapies (eg, antispasmodics, antidepressants, or fibre-based medications); and had had IBS symptoms for 12 months or longer.

Medical exclusion criteria^7^, ^8^ were unexplained rectal bleeding or weight loss, inflammatory bowel disease, coeliac disease, peptic ulcer disease, and colorectal carcinoma. Additionally, patients were excluded if they were younger than 18 years, were unable to participate in CBT because of speech or language difficulties, had no access to an internet-connected computer, had received CBT in the previous 2 years, had had previous access to the web CBT for IBS intervention (Regul8) during the Management of Irritable Bowel Syndrome in Primary Care (MIBS) trial, or were currently participating in another IBS intervention trial.

A patient and public involvement (PPI) representative participated in the trial management group and was included in all phases of trial design, including planning recruitment and recruitment materials. They had been a participant in the MIBS feasibility study^11^ and thus were able to provide first-hand insight into the burden of the intervention and the time required to participate in the research. The CBT interventions were developed with PPI input.^11^ The independent trial steering committee included another PPI member.

Ethical approval was given by the National Research Ethics Service Committee South Central—Berkshire on June 11, 2013 (reference number 13/SC/0206). Additional online informed consent was obtained from participants for the 24-month follow-up.

### Procedures

Two active interventions were assessed in the ACTIB study: therapist-delivered telephone CBT with a detailed patient self-management manual and a low intensity web-based CBT in the form of the Regul8 program developed in the MIBS trial,^11^ with some therapist support. All groups received TAU, with control being TAU alone.

The core CBT content of the two treatment groups was similar, based on an empirical cognitive behavioural model of IBS^12^ and versions of this model tested in previous smaller RCTs.^13^, ^14^ It consisted of education around the brain–gut axis, behavioural techniques to improve bowel habits, developing stable healthy eating and exercise patterns, addressing unhelpful thoughts, managing stress and emotions, focusing on reducing symptoms, and preventing relapse. Treatments were standardised by provision of therapist training and therapist manuals. All therapists were available to work in both therapy groups (telephone-CBT and web-CBT groups) and with any participant regardless of recruitment centre.

Participants randomised to the telephone-CBT group received a detailed self-management CBT manual including homework tasks and recording sheets and were offered six 1-h telephone sessions with a CBT therapist at week 1, 2, 3, 5, 7, and 9. They also received two 1-h booster sessions at 4 months and 8 months (a total of 8 h of therapist support).

Participants in the web-CBT group received three 30-min therapy-support telephone calls at weeks 1, 3, and 5 and two 30-min booster sessions at 4 months and 8 months (2·5 h of therapist support).

TAU was defined as continuation of current medications, which varied from patient to patient, and usual general practitioner or consultant follow-up with no psychological therapy. All general practice or secondary-care sites involved in the study received a hard copy (delivered by the research team or by post) or a digital copy (delivered by email) of the NICE guidance for IBS^3^ to ensure all clinicians had standard best-practice information on IBS management. They also received information to remind them of the guidelines, protocol guidance on prescribing psychological therapies, and inclusion criteria. All participants received a standard information sheet on lifestyle and diet in IBS based on NICE guidance.^3^

At 12 months, information was collected on any changes in IBS treatments and management during the study and numbers of general practitioner and consultant consultations were recorded for all three groups.

After the 12-month follow-up assessments, participants in the TAU group alone were given access to the Regul8 website (but with no therapy support) via an email link; the web-CBT participants also had ongoing access to the Regul8 website. Telephone-CBT participants were not given access to Regul8 but were able to continue to use their CBT manuals. Neither CBT group was offered further therapist support. Thus, the last contact with a trial therapist in the telephone-CBT and web-CBT groups was approximately 16 months before the 24 month follow-up. Participants were free to seek CBT through any available means for IBS or any other condition.

13 trained CBT therapists (10 [77%] of whom were female; mean age 42 years [range 34–52]) based at South London and Maudsley NHS Trust provided the telephone CBT sessions for both therapy groups. Six (46%) of these therapists were clinical psychologists and seven (54%) were cognitive behavioural psychotherapists with a median of 7 years (4–24) experience. All sessions were audio-recorded for supervision and treatment fidelity purposes. Each therapist received a therapist manual, 2-day training, and post-training supervision. Supervision was done in 90-min group sessions every 2 weeks in the first half of the trial, then monthly.

Treatment fidelity was further assessed at the end of trial by two independent experienced CBT therapists using audio-recordings of therapy sessions.

Outcome measures were completed online by participants at 24 months or a paper copy of the questionnaires was posted or telephone follow-up undertaken as described in the protocol^7^ for the 12-month follow-up.^7^

### Outcomes

24-month data were collected on the two co-primary outcomes from the ACTIB trial,^8^ IBS-SSS^10^ and the Work and Social Adjustment Scale (WSAS),^14^ which measures impact of IBS on life including ability to work, manage daily home tasks (eg, cleaning, shopping, cooking, child care, and paying bills), and participate in social activities.

Data were also collected on the ACTIB trial secondary outcome measures at 24 months: Hospital Anxiety and Depression Scale (HADS),^15^ which measures mood as a total distress score; Patient Enablement Questionnaire (PEQ),^16^ which measures people's ability to cope with their illness and life after treatment; and Subject's Global Assessment of Relief (SGA),^17^ which assessed IBS symptom relief in a scale of 1–5.

Participants were also asked whether they had sought CBT for IBS, CBT for any other condition, or had used the web-CBT (ie, the Regul8 website) between the 12-month and 24-month follow-ups.

### Statistical analysis

The statistical analysis of the 24-month outcomes followed the analysis approach used to evaluate the 3-month, 6-month, and 12-month outcomes. Details can be found in the published primary trial publication and online materials.^8^ The analysis of the 24-month data was not prespecified in the original trial protocol as the original trial funding only covered follow-up to 12 months. We sought an extension to the original trial from our funders and the ethics committee and participants were asked to provide consent to collect 24-month data.

Briefly, an intention-to-treat (ITT) approach was used for all 24-month outcomes. For each outcome we compared the CBT groups (telephone-CBT group or web-CBT group) with the TAU group to assess treatment effectiveness. Modelling of continuous variables (IBS-SSS, WSAS, and HADS) relied on normal assumptions for error terms and treatment effects were quantified by trial-group differences and standardised differences (95% CI). The PEQ measure was reclassified as a binary variable with a score of 6 or more considered a responder to facilitate modelling within a logistic regression framework and treatment effects quantified by odds ratios (ORs) with 95% CIs.

Trial groups were compared by multiple imputation with the flexible multivariate imputation by chained equations approach with 100 imputations.^18^ This approach was necessary because we found that non-adherence with treatment was predictive of missing primary outcomes at 12 months in the CBT groups.^8^ We also previously assessed whether baseline variables were predictive of outcome missingness and identified baseline IBS symptom severity score and index of multiple deprivation (IMD)^19^ as further possible predictors.^8^ The linear-regression or logistic-regression analysis models of the multiple-imputation procedure included the respective outcome variable as the dependent variable and trial group (two dummy variables indicating the telephone-CBT and web-CBT groups), baseline values of the outcome (if available), and randomisation stratifier (dummy variables for centres) as explanatory variables. Since both telephone-CBT and web-CBT involved therapists delivering the intervention, possible therapist effects on 24-month outcomes were assessed empirically with the same methods as in the primary trial paper.^8^ Therapist effects were not detected with an α of 10% and so therapist effects were not included in any of the 24-month analysis models. For each outcome variable, the imputation model of the multiple imputation procedure included: all variables of the analysis model; measures of the outcome variable at other assessment timepoints including baseline; and known predictors of missingness (ie, binary adherence variables for telephone-CBT and web-CBT; baseline IBS-SSS; and IMD).

Per-protocol analyses were used to estimate the efficacy of telephone-CBT and web-CBT in terms of primary outcomes of the original 12-month study. The analyses for IBS-SSS and WSAS at 24 months were repeated after restricting the sample to those trial participants who: adhered to the randomised treatment offered to them during the 12-month trial period (adherence to therapy was defined as participants in the web-CBT group completing at least four website sessions and at least one telephone support call or as participants in the telephone-CBT group completing at least four of the initial telephone CBT sessions);^7^, ^8^ did not access any form of CBT during the 12–24-month naturalistic follow-up period; and provided 24-month outcome data (complete-case approach, no imputation). Respective linear regression analyses were adjusted for known baseline predictors of missingness at 12 months (IMD and IBS-SSS).

All analyses were done in Stata, version 14.2.

This study is registered as at the International Standard Randomised Controlled Trial Number registry, number ISRCTN44427879.

### Role of funding source

The funder of the study had no role in study design, data collection, data analysis, data interpretation, or writing of the report. All authors had full access to all of the data and the corresponding author had the final responsibility to submit for publication.

## Results

24-month data collection took place between March 1, 2016, and May 31, 2018. Among 558 patients who were randomly assigned to study groups as part of the ACTIB trial, 323 (58%) provided data at the 24-month follow-up (119 [64%] of 186 in the telephone-CBT group, 99 [54%] of 185 in the web-CBT group, and 105 [56%] of 187 in the TAU group) compared with 391 (70%) at the 12-month follow-up. The mean follow-up time was 638 days (SD 217) and the median was 730 days (IQR 730–730).

Table 1 provides descriptive summaries for outcome measures at baseline, 12 months, and 24 months. We compared the outcomes between each of the CBT groups and the TAU group at 12 months and 24 months (table 2). Although comparisons at 12 months have been presented previously,^8^ the results shown in table 2 differ slightly from those previously published as it was possible to incorporate additional information provided by the 24-month outcomes. This is because multiple imputation makes maximum use of all of the available data and is adjusted for missing data biases to calculate estimated adjusted results. Therefore, including the 24-month data affects the estimates for the whole data set.

**Table 1:** Descriptive summaries for outcome measures at baseline, 12 months, and 24 months

	**Telephone-CBT group**		**Web-CBT group**		**TAU group**		**Overall**	
	Number of patients	Mean (SD) or n (%)	Number of patients	Mean (SD) orn (%)	Number of patients	Mean (SD) or n (%)	Number of patients	Mean (SD) or n (%)
**IBS-SSS**								
Baseline	186	272·3 (95·5)	185	264·2 (99·3)	187	258·5 (91·6)	558	265·0 (95·5)
12 months	136	139·0 (94·8)	124	163·0 (108·8)	131	205·6 (100·5)	391	168·9 (104·8)
24 months	119	164·4 (94·9)	99	167·6 (107·5)	105	197·9 (98·6)	323	176·3 (100·9)
Responders* (12 months)	136	99 (73%)	124	82 (66%)	131	58 (44%)	391	239 (61%)
Responders* (24 months)	119	84 (71%)	99	62 (63%)	105	48 (46%)	323	194 (60%)
**WSAS**								
Baseline	186	12·3 (8·8)	185	13·0 (9·3)	187	12·4 (7·4)	558	12·5 (8·5)
12 months	138	6·0 (7·5)	124	7·4 (7·7)	132	10·8 (9·3)	394	8·1 (8·5)
24 months	118	6·1 (7·6)	99	7·3 (8·0)	105	9·7 (8·5)	322	7·6 (8·1)
**HADS**								
Baseline	186	16·1 (6·9)	185	17·0 (7·3)	187	16·0 (6·4)	558	16·4 (6·9)
12 months	120	12·2 (6·5)	117	12·7 (7·4)	113	15·0 (7·2)	350	13·3 (7·1)
24 months	118	12·1 (6·4)	98	12·2 (7·6)	103	15·1 (6·6)	319	13·1 (7·0)
**PEQ**†								
Non-responders (12 months)	138	30 (22%)	124	56 (45%)	132	101 (77%)	394	187 (47%)
Responders (12 months)	138	108 (78%)	124	68 (55%)	132	31 (23%)	394	207 (53%)
Non-responders (24 months)	119	25 (21%)	99	38 (38%)	103	75 (73%)	321	138 (43%)
Responders (24 months)	119	94 (79%)	99	61 (62%)	103	28 (27%)	321	183 (57%)
**SGA**†								
Non-responders (12 months)	138	21 (15%)	124	31 (25%)	132	77 (58%)	394	129 (33%)
Responders (12 months)	138	117 (85%)	124	93 (75%)	132	55 (42%)	394	265 (67%)
Non-responders (24 months)	45	5 (11%)	35	17 (49%)	44	22 (50%)	124	44 (35%)
Responders (24 months)‡	45	40 (89%)	35	18 (51%)	44	22 (50%)	124	80 (65%)

CBT=cognitive behavioural therapy. TAU=treatment as usual. IBS–SSS=Irritable Bowel Syndrome Symptom Severity Score. WSAS=Work and Social Adjustment Scale. HADS=Hospital Anxiety and Depression Scale. PEQ=Patient Enablement Questionnaire. SGA=Subject's Global Assessment of Relief.

* IBS-SSS responders were defined as participants who had a clinically significant change in IBS-SSS (≥50 points) from baseline to 24 months.

† PEQ responders are defined as patients achieving a score of 6 or more; PEQ was not recorded at baseline.

‡ SGA responders are defined as patients achieving a score of 1–3; SGA was not recorded at baseline and was only recorded at 24 months for participants who completed the outcome measures by paper questionnaire.

**Table 2:** Comparisons between CBT groups and TAU

	**Telephone-CBT *vs* TAU**				**Web-CBT *vs* TAU**			
	Estimated difference or OR* (95% CI)	Test (degrees of freedom)	p value	Standardised difference†	Estimated difference or OR* (95% CI)	Test (degrees of freedom)	p value	Standardised difference†
**IBS-SSS**								
12 months	−62·3 (−90·0 to −34·6)	−4·4 (1981)	p<0·001	0·65	−35·4 (−58·4 to −12·3)	−3·0 (251)	0·003	0·37
24 months	−40·5 (−66·0 to −15·0)	−3·1 (185)	0·002	0·42	−12·9 (−38·8 to 12·9)	−1·0 (178)	0·33	0·14
**WSAS**								
12 months	−3·5 (−5·2 to −1·8)	−4·2 (257)	p<0·001	0·41	−2·9 (−4·5 to −1·3)	−3·5 (287)	p<0·001	0·34
24 months	−3·1 (−4·9 to −1·3)	−3·4 (211)	p<0·001	0·36	−1·9 (−3·7 to −0·1)	−2·1 (213)	0·036	0·22
**HADS**								
12 months	−2·8 (−4·1 to −1·4)	−4·0 (211)	p<0·001	0·40	−2·2 (−3·4 to −0·9)	−3·4 (268)	0·001	0·32
24 months	−3·1 (−4·7 to −1·6)	−4·0 (184)	p<0·001	0·46	−2·7 (−4·4 to −1·0)	−3·1 (142)	0·002	0·39
**PEQ responders**								
12 months‡	9·4 (4·5 to 19·7)	5·9 (2510)	p<0·001	..	3·6 (2·1 to 6·0)	4·7 (775)	p<0·001	..
24 months	8·3 (4·2 to 16·4)	6·1 (359)	p<0·001	..	3·3 (1·8 to 6·0)	3·9 (467)	p<0·001	..

All inferences were derived by multiple imputation as described in the Methods section. Each model used k=100 imputations. CBT=cognitive behavioural therapy. TAU=treatment as usual. IBS–SSS=Irritable Bowel Syndrome Symptom Severity Score. WSAS=Work and Social Adjustment Scale. HADS=Hospital Anxiety and Depression Scale. PEQ=Patient Enablement Questionnaire.

* OR is presented only for PEQ responders.

† Differences were standardised by dividing by the respective baseline SDs for IBS–SSS (95·5), WSAS (8·8), and HADS (6·9).

‡ The 12-month model included therapist effects in the telephone-CBT group, so these effects are conditioned on therapist.

At 24 months, mean IBS-SSS was 40·5 points (95% CI 15·0 to 66·0; p=0·002) lower in the telephone-CBT group and 12·9 points (−12·9 to 38·8; p=0·33) lower in the web-CBT group than in the TAU group, for which mean IBS-SSS was 197·9 (SD 98·6; Table 1, Table 2). At 12 months, the differences between the therapy versus TAU groups were 62·3 points lower (34·6 to 90·0; p<0·001) for the telephone-CBT group and 35·4 lower (12·3 to 58·4; p=0·003) for the web-CBT group. Thus, on the basis of the ITT analysis, a significant difference in mean IBS-SSS was sustained at 24 months for the telephone-CBT group but not the web-CBT group versus the TAU group.

**Figure 1:**
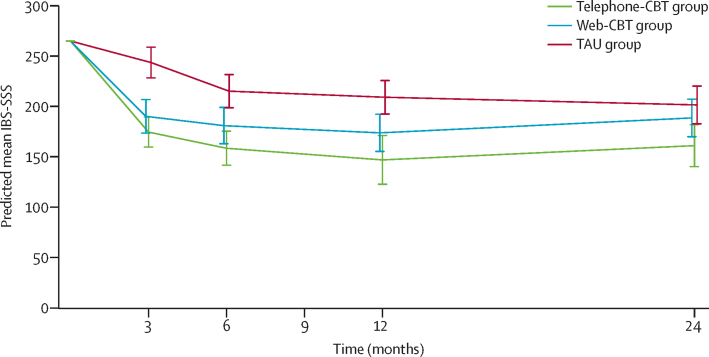
Predicted mean IBS-SSS by assessment time point and trial arm Data are mean (95% CI). Predictions are made from respective analysis models fitted by multiple imputation with baseline set to the sample average value (IBS-SSS=265) and for the site from which most participants were recruited (general practitioners in Southampton). The possible range for IBS-SSS is 0–500. CBT=cognitive behavioural therapy. IBS-SSS=Irritable Bowel Syndrome Symptom Severity Score. TAU=treatment as usual.

We predicted mean IBS-SSS from the multiply imputed data and adjusted for missing-data biases, which allowed us to compare means over time (table 3 and figure 1). At 24 months, mean IBS-SSS had deteriorated by 14·1 points in the telephone-CBT group and by 14·9 points in the web-CBT groups compared with the 12-month follow-up, whereas IBS-SSS in the TAU group had improved by 7·6 points. These trends of both CBT group participants losing previous gains but the TAU group participants improving could possibly explain the observed change in the IBS-SSS differences between the CBT groups and the TAU group at 24 months versus 12 months.

**Table 3:** Predicted change in mean outcomes between 12 months and 24 months

	**12-month follow-up**		**24-month follow-up**		**Predicted change over long–term follow–up period**
	Predicted mean (95% CI) or log odds (95% CI)	Odds (95% CI)	Predicted mean (95% CI) or log odds (95% CI)	Odds (95% CI)	
**IBS-SSS**					
Telephone-CBT group	146·9 (122·7 to 171·0)	..	161·0 (140·2 to 181·9)	..	14·1
Web-CBT group	173·7 (155·3 to 192·2)	..	188·6 (169·9 to 207·2)	..	14·9
TAU group	209·1 (192·5 to 225·8)	..	201·5 (182·7 to 220·3)	..	−7·6
**WSAS**					
Telephone-CBT group	7·2 (6·0 to 8·5)	..	7·0 (5·6 to 8·4)	..	−0·2
Web-CBT group	7·8 (6·6 to 9·1)	..	8·2 (6·9 to 9·5)	..	0·4
TAU group	10·7 (9·5 to 11·9)	..	10·1 (8·8 to 11·4)	..	−0·6
**HADS**					
Telephone-CBT group	12·5 (11·4 to 13·5)	..	12·5 (11·3 to 13·8)	..	0·0
Web-CBT group	13·0 (12·1 to 14·0)	..	13·0 (11·7 to 14·3)	..	0·0
TAU group	15·2 (14·2 to 16·2)	..	15·7 (14·5 to 16·8)	..	0·5
**PEQ**					
Telephone-CBT group	1·08 (0·41 to 1·75)*	2·68 (1·37 to 2.95)	1·11 (0·55 to 1·66)	3·02 (1·73 to 5·28)	0·03†
Web-CBT group	0·11 (−0·29 to 0·51)	1·02 (0·52 to 1·12)	0·18 (−0·24 to 0·60)	1·20 (0·79 to 1·82)	0·07†
TAU group	−1·16 (−1·59 to −0·72)	0·29 (0·15 to 0·31)	−1·01 (−1·47 to −0·55)	0·36 (0·23 to 0·58)	0·15†

Data are predicted mean (95% CI) for IBS-SSS, WSAS, and HADs and log odds (95% CI) and odds (95% CI) for PEQ. Predictions are for sample average values of baseline variables (265 for IBS-SSS, 12·5 for WSAS, and 16·4 for HADS) and for the site from which most participants were recruited (general practices in Southampton). IBS-SSS=Irritable Bowel Syndrome Symptom Severity Score. CBT=cognitive behavioural therapy. TAU=treatment as usual. WSAS=Work and Social Adjustment Scale. HADS=Hospital Anxiety and Depression Scale. PEQ=Patient Enablement Questionnaire.

* For the telephone-CBT group at the 12-month follow-up, therapist effects were found to be significant and were included in the model; the log odds presented here are conditional effects (conditioned on therapist in the telephone-CBT group).

† Difference in log odds.

84 (71%) of 119 patients in the telephone-CBT group, 62 (63%) of 99 in the web-CBT, and 48 (46%) of 105 in the TAU group were IBS-SSS responders (ie, participants who had a clinically significant change in IBS-SSS [≥50 point] from baseline to 24 months; table 1).

**Figure 2:**
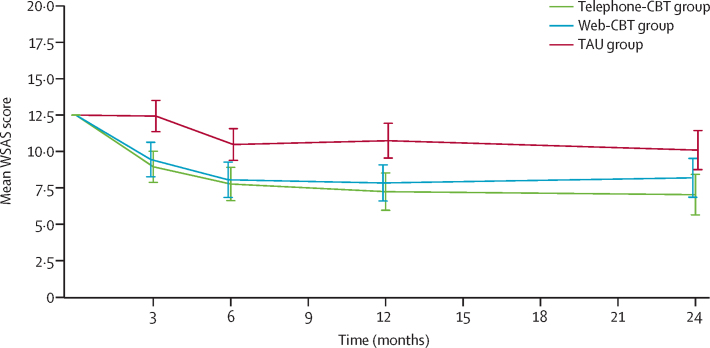
Predicted mean WSAS by assessment time point and trial arm Data are mean (95% CI). Predictions are made from the respective analysis models fitted by multiple imputation with baseline set to the sample average value (WSAS score 12·5) and for the site from which most participants were recruited (general practitioners in Southampton). The possible range for WSAS is 0–40. CBT=Cognitive behavioural therapy. WSAS=Work and Social Adjustment Scale. TAU=treatment as usual.

The WSAS scores maintained significant improvements in both the telephone-CBT and web-CBT groups compared with the TAU group, between 12 months and 24 months. At 24 months, the mean WSAS score was 3·1 points (95% CI 1·3–4·9; p<0·001) lower in the telephone-CBT group and 1·9 points (0·1 to 3·7; p=0·036) lower in the web-CBT group than in the TAU group (mean WSAS score 9·7 [SD 8·5]; Table 1, Table 2). Estimated mean WSAS score in all three groups remained quite stable in the 12–24-month period (figure 2 and table 3).

Further ACTIB outcome measures showed significant improvement in both therapy groups compared with TAU at 24 months (table 2). The mean HADS score was 3·1 points (95% CI 1·6–4·7) lower in the telephone-CBT group (p<0·001) and 2·7 points (1·0–4·4) lower in the web-CBT group (p=0·002) than in the TAU group. For PEQ responders the OR was 8·3 (4·2–16·4; p<0·001) for the telephone-CBT versus the TAU group and 3·3 (1·8–6·0; p=0·001) for the web-CBT versus the TAU group. For SGA there was a problem with data collection at 24 months and too few participants completed this outcome measure for us to formally analyse the data (table 1).

We assessed participants' use of additional CBT treatments during naturalistic follow-up (table 4). 48 (9%) of 558 participants sought any form of CBT for any condition between 12 months and 24 months, of whom 15 (8%) of 186 were in the telephone-CBT group, 13 (7%) of 185 in the web-CBT group, and 20 (11%) of 187 in the TAU group. Eight (1%) of 558 patients sought IBS-specific CBT, of whom three (2%) of 186 were in the telephone-CBT group, one (1%) of 185 in the web-CBT group, and four (2%) of 187 in the TAU group. The proportions of participants seeking CBT for any condition in the 12–24-month period did not differ between groups (Fisher's exact test for telephone-CBT *vs* TAU, p=0·48; for web-CBT *vs* TAU, p=0·27). Only ten (5%) of 187 participants from the TAU group accessed Regul8 despite all TAU participants being sent an access link at 12 months. The percentages of participants deviating from the treatment allocated to them in the trial for any reason (ie, participants who did not receive the allocated treatment or who received some form of CBT in the follow-up period) are shown in table 4.

**Table 4:** Additional CBT treatment sought during naturalistic follow-up

	**Telephone-CBT group (n=186)**	**Web-CBT group (n=185)**	**TAU group (n=187)**	**All (n=558)**
**12–24 months post-trial follow–up period**				
Any CBT sought including Regul8	15 (8%)	13 (7%)	20 (11%)	48 (9%)
CBT for IBS sought*	3 (2%)	1 (1%)	4 (2%)	8 (1%)
CBT for other condition sought*	13 (7%)	12 (6%)	12 (6%)	37 (7%)
Regul8 used*	0 (0%)	0 (0%)	10 (5%)	10 (2%)
**Trial period (0–12 months)**				
Non-adherence with allocated treatment	29 (16%)	57 (31%)	0 (0%)	86 (15%)
**Whole observation period (0–24 months)**				
Participants deviating from the treatment allocated†	43 (23%)	68 (37%)	20 (11%)	131 (23%)

Data are n (%). CBT=cognitive behavioural therapy. IBS=irritable bowel syndrome.

* These treatments are not mutually exclusive. Participants were free to seek treatment for any or all categories.

† Participants who did not receive the allocated treatment or who received some form of CBT in the follow-up period.

We also assessed longer-term efficacy of web-CBT and telephone-CBT via per-protocol analyses. In our per-protocol analysis, mean IBS-SSS at 24 months was 50·0 points (95% CI 24·5–75·5; p<0·001) lower in the telephone-CBT group and 51·5 points (23·9–79·0; p<0·001) lower in the web-CBT group than in the TAU group. Mean WSAS at 24 months 4·1 points (2·2–5·9; p<0·001) lower in the telephone-CBT group and 3·7 points (1·7–5·7; p<0·001) lower in the web-CBT group than in the TAU group. In the per-protocol versus ITT analyses, mean IBS-SSS decreased by an additional 9·5 points in the telephone-CBT group and 38·6 points in the web-CBT group, and the mean WSAS score decreased by additional 1·0 point in the telephone-CBT group and 1·8 points in the web-CBT group, compared with the TAU group. The web-CBT group was subject to higher proportions of non-adherence with therapy than the telephone-CBT group (table 4), which might explain the larger differences found between efficacy (ie, in the per-protocol population) and effectiveness (ie, in the ITT population) for the web-CBT group compared with the telephone-CBT group. However, these efficacy results should be treated with care as they are based on the complete cases at 24 months only and are subject to selection bias, whereas the multiple-imputation effectiveness analyses are able to adjust for further variables driving the missing data generating process.

At the end of the year following the trial, participants were asked to report any adverse events in months 12–24. 41 adverse events were reported (11 in the telephone-CBT group, 15 in the web-CBT group, and 15 in the TAU group). Of these, eight were reported as gastrointestinal-related (two in the telephone-CBT group, four in the web-CBT group, and two in the TAU group), five were psychological (two in the telephone-CBT group, none in the web-CBT group, and three in the TAU group), and six were musculoskeletal (one in the telephone-CBT group, two in the web-CBT group, and three in the TAU group). No adverse events were reported as related to the intervention.

## Discussion

Compared with the TAU group, both telephone-CBT and web-CBT intervention groups showed sustained improvements in outcomes at 24 months. However, these were reduced in magnitude at 24 months versus 12 months. Despite this decrease, the ITT analysis showed that at 24 months both CBT groups reported significantly less impact of IBS on life and the telephone-CBT group reported significantly greater reductions in IBS-SSS than the TAU group. The proportion of patients who had a clinically significant change in IBS symptoms (IBS-SSS change ≥50 points between baseline and 24 months) was 71% in the telephone-CBT group and 63% in the web-based CBT group, compared with 46% in the TAU group. In terms of other outcomes, reductions in total anxiety and depression (HADS) and patient enablement gains (ie, ability to cope with their illness; PEQ) in the CBT arms remained strong at 24 months.

The complete case per-protocol analysis indicated that patients who adhered with the CBT interventions (as defined in our protocol^7^) and did not seek additional CBT treatments in the 12–24-month period maintained significant gains in both IBS-SSS and WSAS outcomes at 24 months in both the CBT groups. The difference in the ITT and per-protocol analysis was particularly marked for the web-CBT group. This difference could be due to the proportion of patients who adhered to the prescribed CBT being somewhat lower in the web-CBT group than that in the telephone-CBT group. Differential adherence between the CBT groups might also explain some of the differences seen in the outcome measures between the CBT groups in the ITT analysis.

This study's strengths include follow-up of a well powered, rigorously conducted RCT with broad inclusion. The IBS-specific CBT was based on an explicit theoretical model^12^, ^20^ informing the detailed patient and therapy manuals and the web-based CBT (Regul8). Therapists were experienced in delivering CBT and trained and supervised to deliver IBS-specific CBT. Trial interventions had good treatment fidelity^8^ and were delivered by NHS therapists in an NHS setting. We believe this is the first large-scale trial of CBT for IBS to publish 24 months follow-up data. This longer-term follow-up provides both patients and clinicians with valuable information that the benefits of CBT for IBS are sustained.

Limitations include the potential for limited external validity as people with IBS who are unwilling to consider undertaking CBT for IBS are unlikely to have participated in the trial. However, we believe that the sample was broadly representative of people with IBS, as the age and gender was similar between those invited to participate in the ACTIB trial and those who were randomly allocated to trial groups, although there was little ethnic diversity. The proportion of patients who participated in follow-up was 58% at 24 months, compared with 70% at 12 months. Thus, there is potential for the 24-month outcome analyses to be affected by missing-data biases. To mitigate against this possibility, multiple imputation, which accommodates all observed predictors of missingness, was used.

The 12-month results from ACTIB^8^ and previous research^6^, ^13^, ^14^ have shown face-to-face and telephone-delivered CBT to be beneficial for IBS, particularly immediately after completing treatment. However, a Cochrane review^6^ concluded that it was unclear whether the effects were maintained in the longer term. Other large published trials^4^, ^5^ of CBT for IBS report 6 months post-randomisation follow-up. For instance, a three-arm RCT^5^ (n=436) compared face-to-face CBT for IBS with home-based CBT (minimal-contact CBT) using self-study materials and IBS education alone.^5^ This study showed promising results for low-intensity CBT for IBS. Improvement was reported at 2 weeks in both their CBT arms compared with education alone, and on gastroenterologist (but not patient) ratings at 6 months on the clinical global impressions improvement scale. However, follow-up was limited to 6 months and participants were only recruited from tertiary centres and did not show significantly greater improvements for CBT than education alone on IBS-SSS at any timepoint. ACTIB 24-month follow-up showed improvements in IBS-SSS and global symptoms (SGA). The gains maintained at 24 months are despite having no further input from a trial therapist since month 8 (ie, 16 months before the 24-month outcome measures were recorded). Most of the therapy contact was in the first 3 months (two booster sessions were offered at 4 and 8 months) and the overall maximum dose of therapy was 8 h for the telephone-CBT group and 2·5 h for the web-CBT group. In the NHS, therapist-delivered CBT is typically offered as 5–20 sessions, so even the higher intensity CBT is at the lower end of the typical therapy dose.

Currently, clinicians have few options to offer people with refractory IBS, particularly in primary care. This study shows that IBS-specific CBT has the potential to provide significant improvement in terms of IBS's impact on life and symptom severity, with ongoing benefits at 24 months. Offering both web-CBT and telephone-CBT in NHS services such as Improving Access to Psychological Therapy could allow many patients to gain substantial benefits with web-CBT with minimal therapist input while allowing a step-up approach to telephone-CBT for those needing additional support.

We are planning a future publication on the cost effectiveness of telephone-delivered and web-based CBT for IBS interventions and also on whether there are identifiable moderators and mediators that would indicate who is most likely to benefit from CBT for IBS. Further research is needed to assess whether telephone-delivered and web-based CBT can be widely disseminated in a non-trial clinical settings.

This study reinforces NICE guidance^3^ that patients with refractory IBS should be offered CBT for IBS, which is currently not widely available. Our results show that both telephone therapist-delivered and web-based CBT for IBS can provide long-term benefits.

## Data sharing

HAE can be contacted at the corresponding email address regarding data-sharing requests, including access to the patient self-management, therapist training, and therapist manuals. Individual participant data that underlie the results reported in this Article might be available after de-identification to researchers who provide a methodologically sound proposal and whose proposed use of the data has been approved by an independent review committee. To gain access, data requesters will need to sign a data access agreement. The CBT patient and therapist manuals used in the telephone-CBT group are freely available on request to the corresponding author. The patient manual is background intellectual property developed by RMM and TC in previous work. The therapist manual was developed for the ACTIB trial. These manuals were only made available once the 12-month ACTIB follow up was complete.
